# Topological Equivalence Theorem and Double-Copy for Chern–Simons Scattering Amplitudes

**DOI:** 10.34133/research.0072

**Published:** 2023-03-17

**Authors:** Yan-Feng Hang, Hong-Jian He, Cong Shen

**Affiliations:** ^1^ Tsung-Dao Lee Institute and School of Physics and Astronomy, Key Laboratory for Particle Astrophysics and Cosmology (MOE), Shanghai Key Laboratory for Particle Physics and Cosmology, Shanghai Jiao Tong University, Shanghai, China.; ^2^ Department of Physics and Institute of Modern Physics, Tsinghua University, Beijing, China.; ^3^ Center for High Energy Physics, Peking University, Beijing, China.; ^4^ Fields and Strings Laboratory, Institute of Physics, Ecole Polytechnique Federale de Lausanne, Lausanne, Switzerland.

## Abstract

We study the mechanism of topological mass generation for 3-dimensional Chern–Simons gauge theories and propose a brand-new topological equivalence theorem to connect scattering amplitudes of the physical gauge boson states to that of the transverse states under high-energy expansion. We prove a general energy cancelation mechanism for *N*-point physical gauge boson amplitudes, which predicts large cancelations of *E*^4 − *L*^ → *E*^(4 − *L*) − *N*^ at any *L*-loop level (*L* ⩾ 0). We extend the double-copy approach to construct massive graviton amplitudes and to study their structures. We newly uncovered a series of strikingly large energy cancelations *E*^12^ → *E*^1^ of the tree-level 4-graviton scattering amplitude under high-energy expansion and establish a new correspondence between the 2 energy cancelations in the topologically massive Yang–Mills gauge theory and the topologically massive gravity theory. We further study the scattering amplitudes of Chern–Simons gauge bosons and gravitons in the nonrelativistic limit.

## Introduction

The (2+1)-dimensional (3D) Chern–Simons (CS) theories naturally realize gauge-invariant (diffeomorphism-invariant) topological mass terms for gauge bosons and gravitons [1,2]. Understanding the underlying mechanism of such topological mass generations and how it determines the structure of the massive gauge boson/graviton scattering amplitudes is important for applying the modern quantum field theories to particle physics and condensed matter physics [1–4].

In this work, we study the dynamics of topological mass generation for the 3D CS gauge and gravity theories [1,2]. The 3D gauge fields can acquire gauge-invariant topological mass terms à la Chern–Simons [5] and without invoking the conventional Higgs mechanism [6–10]. Adding the 3D CS term will convert the transverse polarization state of massless gauge boson into the physical polarization state of massive gauge boson, which conserves the physical degree of freedom of a gauge boson: 1 = 1. For this, we propose a conceptually new topological equivalence theorem (TET) to formulate the topological mass generation at *S*-matrix level, which quantitatively connects the scattering amplitudes of the physical polarization states of massive gauge bosons to that of the corresponding transverse gauge bosons. This differs essentially from the conventional equivalence theorem (ET) [11] of the 4D standard model and from the Kaluza–Klein (KK) ET for the compactified 5D gauge theories [12–15] and for the compactified 5D general relativity [16,17].

We newly develop a general 3D power counting method to count the leading energy dependence of scattering amplitudes in both the topologically massive Yang–Mills (TMYM) theory and topologically massive gravity (TMG) theory. Using the TET identity and power counting method for TMYM theories, we uncover nontrivial energy cancelations among individual diagrams in the tree-level *N*-gauge boson amplitudes, *E*^4^ → *E*^4 − *N*^, for *N* ⩾ 4. We will demonstrate that the TET provides a general mechanism to guarantee such highly nontrivial energy cancelations for the 3D massive gauge boson scattering amplitudes and the 3D massive graviton scattering amplitudes (through the double-copy construction).

With these, we extend the conventional double-copy approach and construct the massive 4-graviton amplitudes of the TMG theory from the corresponding 4-gauge boson amplitudes of the TMYM theory. The conventional double-copy method of Bern–Carrasco–Johansson [18–20] applies to massless gauge/gravity theories and was inspired by the Kawai–Lewellen–Tye [21,22] relation that connects the product of open-string amplitudes to that of the closed string at tree level. Some recent works attempted to extend the double-copy method to the 4D massive YM versus Fierz–Pauli-like massive gravity [23–26], to the KK-inspired effective gauge theory with extra global U(1) [27], to the compactified 5D KK gauge/gravity theories [16,17,28], and to the compactified KK bosonic string theory [29]. There are also studies on the double copy of 3D supersymmetric CS theories in the massless limit [30,31]. The recent double-copy studies include the 3D CS gauge theories with or without matter fields and the 3D covariant color–kinematics duality [32–35].

Our extended double-copy construction of the 3D massive 4-graviton amplitude from the 3D massive 4-gauge boson amplitude at tree level demonstrates strikingly large energy cancelations, *E*^12^ → *E*^1^*,* in the high-energy graviton amplitude. With these, we establish a new correspondence between the 2 types of distinctive energy cancelations in the massive gauge boson amplitudes and the massive graviton amplitudes: *E*^4^ → *E*^0^ in the TMYM theory and *E*^12^ → *E*^1^ in the TMG theory. Finally, for possible applications to the condensed matter system, we further study the scattering amplitudes of CS gauge bosons and gravitons in the nonrelativistic limit.

## Topological Mass Generation for CS Gauge Theories

The 3D Abelian and non-Abelian CS gauge theories may be called the topologically massive quantum electrodynamics (TMQED) and the TMYM, respectively. Their Lagrangians take the following forms:

$$\begin{array}{c} \;L_{\text{TMQED}}=-\frac{\;1\;}{4}\;F_{\mu \nu }^{2}+\frac{\;1\;}{2}\;\tilde{m}\;\varepsilon^{\mu \nu \rho }A_{\mu }\partial_{\nu }A_{\rho }, \end{array}$$ (1A)

$$\begin{array}{c} \;L_{\text{TMYM}}=-\frac{\;1\;}{2}\;\mathrm{tr}F_{\mu \nu }^{2}+\tilde{m}\varepsilon^{\mu \nu \rho }\mathrm{tr}\left[A_{\mu }\left(\partial_{\nu }-\frac{\;2ig\;}{3}A_{\nu }\right)A_{\rho }\right], \end{array}$$ (1B)

where $\left(F_{\mu \nu },A_{\mu }\right)=\left(F_{\mu \nu }^{a},\;A_{\mu }^{a}\right)T^{a}$ and *T^a^* denotes the generator of the SU(*N*) gauge group. The matter fields can be further added to the above Lagrangians when needed. We note that, in Eq. 1A and B, the gauge bosons acquire a topological mass $m=|\tilde{m}|$ from the CS term, where the ratio $s=\tilde{m}/m=\pm 1$ corresponds to their spin [1–3]. Under a general gauge transformation, the action of TMQED theory is invariant up to a trivial surface term. While for the TMYM theory, the change of its action will contribute to a phase factor *e*^i2*πwn*^, where *w* ∈ ℤ represents the winding number that follows from the homotopy group Π_3_[SU(*N*)] ≅ ℤ [4], and *n* is the CS level $n=4\pi \tilde{m}/g^{2}\in \mathbb{Z}$ . This ensures the phase factor *e*^i2*πwn*^ = 1.

The on-shell gauge field has the plane–wave solution $A_{\mu }^{a}=c^{a}\epsilon_{\mu }\left(p\right)e^{-ip\cdot x}\;$ from the equation of motion, where $p^{\mu }\;A_{\mu }^{a}=0$. Thus, the polarization vector obeys the following equation of motion:

$$\left(m\eta^{\mu \nu }-is\varepsilon^{\mu \rho \nu }p_{\rho }\right)\;\epsilon_{\nu }\left(p\right)=0.$$ (2)

Because the CS term does not add any new field, the physical degrees of freedom of each gauge field $A_{\mu }^{a}$ is conserved before and after setting the *m* = 0 limit [36,37], i.e., 1 = 1. The conservation of the physical degrees of freedom of $A_{\mu }^{a}$ can be further understood from analyzing the (2+1)D little group [38,39].

A 3D massive gauge boson in the rest frame has momentum ${\bar{p}}^{\mu }=\left(m,0,0\right)$, and its physical polarization vector is solved as $\epsilon_{P}^{\mu }\left(\bar{p}\right)=\frac{\;1\;}{\sqrt{2\;}\;}\;\left(0,1,-is\right)$. The $\epsilon_{P}^{\mu }\left(\bar{p}\right)$ can be boosted to $\epsilon_{P}^{\mu }\left(p\right)$ for a general momentum *p^μ^* = *E*(1, *βs_θ_*, *βc_θ_*) [39]. We find that $\epsilon_{P}^{\mu }\left(p\right)$ can be generally decomposed as:

$$\epsilon_{P}^{\mu }=\frac{\;1\;}{\sqrt{2\;}\;}\;\epsilon_{T}^{\mu }+\epsilon_{L}^{\mu },$$ (3A)

$$\begin{array}{c} \epsilon_{T}^{\mu }=(0,isc_{\theta },-iss_{\theta }),\;\epsilon_{L}^{\mu }=\bar{E}(\beta ,s_{\theta },c_{\theta }), \end{array}$$ (3B)

where $\epsilon_{T}^{\mu }$ ($\epsilon_{L}^{\mu }$) denotes the transverse (longitudinal) polarization vector, $\beta ={\left(1-{\bar{E}}^{-2}\right)}^{1/2}$, $\bar{E}=E/m$, and (*s_θ_*, *c_θ_*) =(sin*θ*, cos*θ*). Hence, using Eq. 3A and B, we can define the on-shell polarization states of the gauge field $A_{\mu }^{a}$:

$$A_{P}^{a}=\epsilon_{P}^{\mu }A_{\mu }^{a}=\frac{\;1\;}{\sqrt{2\;}\;}\;A_{T}^{a}+A_{L}^{a},$$ (4A)

$$A_{X}^{a}=\epsilon_{X}^{\mu }A_{\mu }^{a}=\frac{\;1\;}{\sqrt{2\;}\;}\;A_{T}^{a}+A_{L}^{a},$$ (4B)

$$A_{S}^{a}=\epsilon_{S}^{\mu }A_{\mu }^{a}=\left(p^{\mu }/m\right)A_{\mu }^{a},$$ (4C)

where $\epsilon_{S}^{\mu }=p^{\mu }/m=\epsilon_{L}^{\mu }-v^{\mu }$ with the residual term $v^{\mu }=O\left(m/E\right)$, and $\epsilon_{P}\cdot \epsilon_{X}^{*}=\epsilon_{P}\cdot \epsilon_{S}^{*}=\epsilon_{X}\cdot \epsilon_{S}^{*}=0$. We note that the 3D massive gauge boson $A_{\mu }^{a}$ has 3 possible states in total, including 1 physical polarization state $A_{P}^{a}$ and 2 unphysical polarization states ($A_{X}^{a},A_{S}^{a}$). In contrast, the massless gauge boson contains 1 physical transverse polarization $A_{T}^{a}$ and 2 unphysical polarizations $\left(A_{L}^{a},\;A_{S}^{a}\right)$ with $\epsilon_{L}^{\mu }+\epsilon_{S}^{\mu }\propto p^{\mu }$. We observe that adding the CS term for $A_{\mu }^{a}$ field dynamically generates a new massive physical state $A_{P}^{a}$ and converts its orthogonal combination $A_{X}^{a}$ into an unphysical state, whereas the scalar polarization state $A_{S}^{a}$ remains unphysical because it appears in the function $F^{a}\propto A_{S}^{a}$ of the gauge-fixing term:

$$L_{\mathrm{GF}}=-\frac{\;1\;}{2\xi }\;{\left(F^{a}\right)}^{2},\;F^{a}=\partial^{\mu }\;A_{\mu }^{a}.$$ (5)

We stress that one cannot naively take massless limit *m* → 0 for the massive CS theory because it causes the polarization vector $\epsilon_{L}^{\mu }\propto E/m\to \infty$ and thus $\epsilon_{P}^{\mu }\to \infty$, which makes the physical state $A_{P}^{a}\to \infty$ and thus ill-defined*.* Hence, the current analysis of the dynamics of the massive CS theory is highly nontrivial, from which we will establish a brand-new TET in the next section.

## Formulation of the TET

The CS action from Eq. 1A and B is gauge-invariant, and using the method of [11,40,41], we can derive a Slavnov–Taylor-type identity:

$$\langle 0|F^{a_{1}}\left(p_{1}\right)\cdots F^{a_{N}}\left(p_{N}\right)\Phi |0\rangle =0,$$ (6)

where  $F^{a}\left(p_{j}\right)=\;-ip_{j}^{\mu }\;A_{\mu }^{a}$  and the symbol Φ denotes any other on-shell physical fields after the Lehmann–Symanzik–Zimmermann (LSZ) amputation. Because the function $F^{a}$ contains only one single-gauge field $A_{\mu }^{a}$, it is straightforward to amputate each external $F^{a}$ line by the LSZ reduction, where we impose the on-shell condition $p_{j}^{2}=-m^{2}$ for each external line. From Eq. 3A and the relation $\epsilon_{S}^{\mu }=\epsilon_{L}^{\mu }-v^{\mu }$ with the residual term $v^{\mu }=O\left(m/E\right)$, we deduce the following identity:

$$\epsilon_{S}^{\mu }=\sqrt{2}\epsilon_{P}^{\mu }-\left(\epsilon_{T}^{\mu }+v^{\mu }\right),$$ (7)

Using Eqs. 4C and 7, we can reexpress the gauge-fixing function $F^{a}\left(p\right)=-imA_{S}^{a}\;$ as follows:

$$\begin{array}{c} F^{a}\left(p\right)=-i\sqrt{2}m\left(A_{P}^{a}-\Omega^{a}\right),\;\Omega^{a}=\frac{\;1\;}{\sqrt{2\;}\;}\;\left(A_{T}^{a}+v^{a}\right), \end{array}$$ (8)

where $v^{a}=v^{\mu }A_{\mu }^{a}$ . Making the LSZ reduction on Eq. 6 and combining it with Eq. 8, we derive the following TET identity for the scattering amplitudes:

$$\begin{array}{c} T[A_{P}^{a_{1}},\cdots ,A_{P}^{a_{N}},\Phi ]=T[{\tilde{A}}_{T}^{a_{1}},\cdots ,{\tilde{A}}_{T}^{a_{N}},\Phi ]+T_{v}, \end{array}$$ (9A)

$$\begin{array}{c} T_{v}=\sum_{j=1}^{N}‍T[{\tilde{v}}^{a_{1}},\cdots ,{\tilde{v}}^{a_{j}},{\tilde{A}}_{T}^{a_{j+1}},\cdots ,{\tilde{A}}_{T}^{a_{N}},\Phi ], \end{array}$$ (9B)

where ${\tilde{A}}_{T}^{a}=\frac{\;1\;}{\sqrt{2\;}\;}\;A_{T}^{a}\;$ and ${\tilde{v}}^{a}=\frac{\;1\;}{\sqrt{2\;}\;}\;v^{a}$. In the above, the residual term $T_{v}$ is suppressed by the factor $v^{\mu }=O\left(m/E\right)\ll 1$ under high-energy expansion. The TET identity (Eq. 9A) states that the $A_{P}^{a}$ amplitude equals the corresponding $A_{T}^{a}$ amplitude in the high-energy limit. We also observe that the right-hand side (RHS) of Eq. 9A receives no multiplicative modification factor at loop level, because both $A_{P}^{a}$ and $A_{T}^{a}$ belong to the same gauge field $A_{\mu }^{a}$. This feature differs from the conventional ET [40–42] for the Higgs mechanism of the standard model.

Generalizing the previous power-counting method in 4D theories [43,44] and in 5D theories [16,17], we derive a new power-counting rule for the 3D CS gauge theories. For a given amplitude, we count the energy dependence with the power:

$$\begin{array}{c} D_{E}=\left(E_{A_{P}}-E_{v}\right)+\left(4-E-{\bar{V}}_{3}\right)-L, \end{array}$$ (10)

where $\left(E,E_{A_{P}},E_{v}\right)$ denote the numbers of the external lines, the external physical states $A_{P}^{a}$ , and the external states with *v^μ^* factor, respectively. The ${\bar{V}}_{3}$ is the number of cubic vertices containing no derivative (which arise from the non-Abelian CS term), and *L* stands for the number of loops. For the scattering amplitudes of pure gauge bosons ($A_{P}^{a}$) with the number of external $A_{P}^{a}$ states $E=E_{A_{P}}=N$ and $E_{v}=0\;$, we can use Eq. 10 to deduce its leading individual contributions to be of $O\left(E^{4}\right)$ at tree level. For the scattering amplitudes of pure $A_{T}^{a}$ gauge bosons with the number of external $A_{T}^{a}$ states $E=E_{A_{T}}=N$ and $E_{A_{P}}=E_{v}=0$, its individual leading contributions scale like $O\left(E^{4-N}\right)$. With these, our TET identity (Eq. 9A) guarantees the energy cancelation in the *N*-gauge boson ($A_{P}^{a}$) scattering amplitude on its left-hand side: *E*^4^ → *E*^4 − *N*^. This is because, on the RHS of Eq. 9A, the pure *N*-gauge boson $A_{T}^{a}$ amplitude scales as $O\left(E^{4-N}\right)$ and the residual term $T_{v}$ (with $E_{v}⩾1$) scales no more than $O\left(E^{3-N}\right)$. We can readily generalize this result up to *L*-loop level and deduce the following energy power cancelations:

$$\Delta D_{E}=D_{E}\left[N\;A_{P}^{a}\right]-D_{E}\left[N\;A_{T}^{a}\right]=N.$$ (11)

For the sake of later analysis, we also give the power counting rule on the high-energy leading *E* dependence of graviton scattering amplitudes in the TMG theory [39]:

$$D_{E}=2E_{h_{P}}+\left(2+V_{d3}+L\right),$$ (12)

where $V_{d3}$ denotes the number of vertices containing 3 partial derivatives coming from the gravitational CS term in Eq. 17, and $E_{h_{P}}$ denotes the number of external physical graviton states *h*_P_.

## Massive Gauge Boson Amplitudes and Energy Cancelations

In this section, we compute explicitly the 4-gauge boson scattering amplitudes $T\left[A_{P}^{a}A_{P}^{b}\to A_{P}^{c}A_{P}^{d}\right]\left(\equiv T\left[4A_{P}^{a}\right]\right)$ and $T\left[A_{T}^{a}A_{T}^{b}\to A_{T}^{c}A_{T}^{d}\right]\left(\equiv T\left[4A_{T}^{a}\right]\right)$ in the 3D TMYM theory. They receive contributions from the contact diagram and the pole diagrams via (*s*, *t*, *u*) channels, as shown in the first row of Figure. Using the power counting rule (Eq. 10), we deduce that the high-energy leading contributions of $T\left[4A_{P}^{a}\right]$ and $T[4A_{T}^{a}]$ scale like *E*^4^ and *E*^0^, respectively. Hence, using the TET identity (Eq. 9A), we would predict the exact energy cancelations at $O(E^{4},E^{3},E^{2},E^{1})$ in the physical gauge boson amplitude $T\left[4A_{P}^{a}\right]$, because it should match to the leading energy dependence of $T\left[4A_{T}^{a}\right]$ on the RHS of the TET identity (Eq. 9A).

**Figure.**
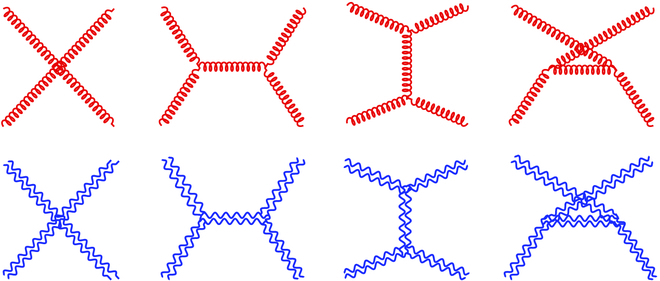
Feynman diagrams in the first row (red color) contribute to the 4-gauge boson scattering processes $A_{P}^{a}A_{P}^{b}\to A_{P}^{c}A_{P}^{d}$ and $A_{T}^{a}A_{T}^{b}\to A_{T}^{c}A_{T}^{d}$ in the TMYM theory, whereas Feynman diagrams in the second row (blue color) contribute to the 4-graviton scattering process *h*_P_*h*_P_ → *h*_P_*h*_P_ in the TMG theory. Both the gauge boson and graviton scattering processes contain the contributions from the contact diagrams and the (*s*, *t*, *u*) channels.

Then, we compute the full 4-point $A_{P}^{a}$ amplitude at tree level and present it in the following compact form:

$$\begin{array}{c} T\left[4A_{P}^{a}\right]=g^{2}\;\left(\frac{\;C_{s}\;N_{s}\;}{\;s-m^{2}\;}+\frac{\;C_{t}\;N_{t}\;}{\;t-m^{2}\;}+\frac{\;C_{u}\;N_{u}\;}{\;u-m^{2}\;}\right), \end{array}$$ (13)

where the color factors $\begin{array}{c} \left(\;C_{s},\;C_{t},\;C_{u}\;\right)\;=\;\left(\;C^{\mathrm{abe}}\;C^{\mathrm{cde}},\;C^{\mathrm{ade}}\;C^{\mathrm{bce}},\;C^{\mathrm{ace}}\;C^{\mathrm{dbe}}\;\right) \end{array}$ and the explicit expressions of kinematic numerators $\left(N_{s},N_{t},N_{u}\right)$ are given in the Supplementary Material [39]. We make high-energy expansion of the full $A_{P}^{a}$ amplitude in terms of $1/\bar{s}$ or $1/{\bar{s}}_{0}$, where $\bar{s}=s/m^{2}$, ${\bar{s}}_{0}=s_{0}/m^{2}$, and *s*_0_ = *s* − 4*m*^2^. Thus, we can explicitly demonstrate the exact energy cancelations at each order of *E^n^* (*n* = 4, 3, 2, 1), which are summarized in Table 1. We find that the $O\left(E^{4}\right)$ contributions cancel exactly between the contact diagram and the pole diagram in each channel of *j* = *s*, *t*, *u*. The sum of each $O\left(E^{n}\right)$ contributions (with *n* = 3, 2, 1) cancels exactly because of the Jacobi identity holds, $C_{s}+C_{t}+C_{u}=0$. For comparison, we have performed a parallel analysis of the exact energy cancelations at $O\left(E^{n}\right)$ (with *n* = 4, 3, 2, 1) under the high-energy expansion of $1/{\bar{s}}_{0}$, which are summarized in the Supplementary Material [39].

**Table 1.** Energy cancelations for amplitude $T\left[4A_{P}^{a}\right]=T_{c}+T_{s}+T_{t}+T_{u}$ in the 3D TMYM theory, where the contribution of the contact channel is decomposed into 3 sub-amplitudes according to the color factors, $T_{c}=T_{\mathrm{cs}}+T_{\mathrm{ct}}+T_{\mathrm{cu}}$. The energy factors are $\bar{s}=s/m^{2}=4{\bar{E}}^{2}$ and $\bar{E}=E/m\;$, whereas for the angular dependence the notations are (*s_nθ_*, *c_nθ_*) = (sin *nθ*, cos *nθ*). A common overall factor (*g*^2^/128) in each amplitude is not displayed for simplicity.

Amplitude	$\times {\bar{s}}^{2}$	$\times {\bar{s}}^{3/2}$	$\times \bar{s}$	$\times {\bar{s}}^{1/2}$
$T_{\mathrm{cs}}$	$8s_{\theta }\;C_{s}$	$i32s_{\theta }C_{s}$	0	$-i128s_{\theta }\;C_{s}$
$T_{\mathrm{ct}}$	$-\left(5+4c_{\theta }-c_{2\theta }\right)\;C_{t}$	$-i8\left(2s_{\theta }-s_{2\theta }\right)\;C_{t}$	$8\left(5+3c_{2\theta }\right)\;C_{t}$	$i32\left(2s_{\theta }+s_{2\theta }\right)\;C_{t}$
$T_{\mathrm{cu}}$	$\left(5-4c_{\theta }-c_{2\theta }\right)\;C_{u}$	$-i8\left(2s_{\theta }+s_{2\theta }\right)\;C_{u}$	$-8\left(5+3c_{2\theta }\right)\;C_{u}$	$i32\left(2s_{\theta }-s_{2\theta }\right)\;C_{u}$
$T_{s}$	$-8s_{\theta }\;C_{s}$	$-i56s_{\theta }\;C_{s}$	$-128c_{\theta }\;C_{s}$	$-i32s_{\theta }\;C_{s}$
$T_{t}$	$\left(5+4c_{\theta }-c_{2\theta }\right)\;C_{t}$	$-i8\left(s_{\theta }+s_{2\theta }\right)\;C_{t}$	$-8\left(5+16c_{\theta }+3c_{2\theta }\right)\;C_{t}$	$-i32\left(7s_{\theta }+s_{2\theta }\right)\;C_{t}$
$T_{u}$	$-\left(5-4c_{\theta }-c_{2\theta }\right)\;C_{u}$	$-i8\left(s_{\theta }-s_{2\theta }\right)\;C_{u}$	$8\left(5-16c_{\theta }+3c_{2\theta }\right)\;C_{u}$	$-i32\left(7s_{\theta }-s_{2\theta }\right)\;C_{u}$
Sum	0	0	0	0

After all the high-energy cancelations, we systematically derive the leading nonzero scattering amplitudes of $T\left[4A_{P}^{a}\right]$ and $T\left[4A_{T}^{a}\right]$ at $O\left(E^{0}\right)$ under the $1/\bar{s}$ expansion, which take the following forms:

$$\begin{array}{c} \begin{array}{l} T_{0}\left[4A_{P}^{a}\right]=g^{2}\left[C_{s}\frac{7c_{\theta }}{4}+C_{t}\frac{7+7c_{\theta }+4c_{2\theta }}{4\left(1+c_{\theta }\right)}+C_{u}\frac{-\left(7-7c_{\theta }+4c_{2\theta }\right)}{4\left(1-c_{\theta }\right)}\right],\\ T_{0}\left[4A_{T}^{a}\right]=g^{2}\left[C_{s}\frac{-c_{\theta }}{4}+C_{t}\frac{3-c_{\theta }}{4\left(1+c_{\theta }\right)}+C_{u}\frac{-\left(3+c_{\theta }\right)}{4\left(1-c_{\theta }\right)}\right], \end{array} \end{array}$$ (14)

where (*c_θ_*, *c*_2*θ*_) = (cos *θ*, cos2*θ*). These 2 amplitudes differ by an amount: $\begin{array}{c} T_{0}\left[4A_{P}^{a}\right]\;-\;T_{0}\left[4A_{T}^{a}\right]\;=\;2c_{\theta }g^{2}\left(C_{s}+C_{t}+C_{u}\right)\;=\;0 \end{array}$  , which vanishes identically because of the Jacobi identity. Hence, this demonstrates explicitly that the TET (Eq. 9A and B) holds in the high-energy limit. For comparison, we further derive the leading nonzero gauge boson scattering amplitudes at $O\left(E^{0}\right)$ under the $1/{\bar{s}}_{0}$ expansion:

$$\begin{array}{c} \begin{array}{l} T_{0}^{\prime }\left[4A_{P}^{a}\right]=g^{2}\left[C_{s}\frac{-9c_{\theta }}{4}+C_{t}\frac{-\left(1+9c_{\theta }+4c_{2\theta }\right)}{4\left(1+c_{\theta }\right)}+C_{u}\frac{1-9c_{\theta }+4c_{2\theta }}{4\left(1-c_{\theta }\right)}\right],\\ T_{0}^{\prime }\left[4A_{P}^{a}\right]=g^{2}\;\left[\;C_{s}\frac{-c_{\theta }}{4}+C_{t}\frac{3-c_{\theta }}{\;4\left(1+c_{\theta }\right)\;}+C_{u}\frac{-\left(3+c_{\theta }\right)\;}{\;4\left(1-c_{\theta }\right)\;}\right]. \end{array} \end{array}$$ (15)

Inspecting Eqs. 14 and 15, we find that the 2 amplitudes of transverse gauge bosons are equal, $T_{0}\left[4A_{T}^{a}\right]=T_{0}^{\prime }\left[4A_{T}^{a}\right]$, whereas the 2 amplitudes of physical gauge bosons has a difference $\begin{array}{c} T_{0}^{\prime }\left[4A_{P}^{a}\right]-T_{0}\left[4A_{P}^{a}\right]=4c_{\theta }g^{2}\left(C_{s}+C_{t}+C_{u}\right)=0 \end{array}$, which vanishes identically because of the Jacobi identity. Hence, the leading nonzero amplitudes of $O\left(E^{0}\right)$ are universal and independent of the high-energy expansion parameters (either $1/\bar{s}$ or $1/{\bar{s}}_{0}$).

From the above analysis, we have well understood the structure of the 4-gauge boson scattering amplitude (Eq. 13) in the ultraviolet (UV) region. We have justified its energy cancelations order by order under the high-energy expansion, at each $O\left(E^{n}\right)$ with *n* = 4, 3, 2, 1, and have proved explicitly the TET (Eq. 9A) at $O\left(E^{0}\right)$.

Next, for possible applications to the condensed matter system and other low-energy studies, we further analyze the nonrelativistic limit and make the low-energy expansion of the 4-point gauge boson scattering amplitudes (Eq. 13). Thus, we derive the following expanded scattering amplitudes of gauge bosons at the leading order (LO) and next-to-leading order (NLO) of the low-energy expansion:

$$\begin{array}{c} \begin{array}{l} T_{0}=\frac{\;-ig^{2}}{\;4s_{\theta }\;}e^{i2\theta }\left\{C_{s}2\left[\left(3-5c_{2\theta }\right)+i5s_{2\theta }\right]\right.\\ \;+C_{t}\left[\left(17-2c_{\theta }-19c_{2\theta }\right)+i\left(66s_{\theta }+35s_{2\theta }\right)\right]\\ \left.\;+C_{u}\left[\left(17+2c_{\theta }-19c_{2\theta }\right)-i\left(66s_{\theta }-35s_{2\theta }\right)\right]\right\}, \end{array} \end{array}$$ (16A)

$$\begin{array}{c} \begin{array}{l} \;\delta T=-\frac{\;ig^{2}s\;}{\;16m^{2}s_{\theta }\;}e^{i2\theta }\left\{C_{s}2\left[\left(-3+5c_{2\theta }\right)-i5s_{2\theta }\right]\right.\\ \;+C_{t}\left[-\left(13+2c_{\theta }-19c_{2\theta }\right)-i\left(46s_{\theta }+35s_{2\theta }\right)\right]\\ \;+\left.C_{u}\left[-\left(13-2c_{\theta }-19c_{2\theta }\right)+i\left(46s_{\theta }-35s_{2\theta }\right)\right]\right\}. \end{array} \end{array}$$ (16B)

We see that, under the nonrelativistic expansion at low energies, the LO scattering amplitude $T_{0}$ of the physical gauge bosons scales as *E*^0^ and the NLO amplitude δT scales as *E*^2^/*m*^2^.

## Constructing Graviton Scattering Amplitude from Double Copy

The conventional Einstein gravity in 3D has no physical content [1,2,45–49], whereas the TMG includes the gravitational CS action with which the graviton becomes massive and acquires a physical polarization state *h*_P_. The CS action of the TMG theory $\int \;‍d^{3}x\;L_{\mathrm{TMG}}$ contains the Lagrangian:

$$\begin{array}{c} L_{\mathrm{TMG}}=\frac{\;-2\;}{\;\kappa^{2}\;}\;\left[\;\sqrt{-g}R-\frac{\varepsilon^{\mu \nu \rho }}{2\tilde{m}}\Gamma_{\rho \beta }^{\alpha }\left(\partial_{\mu }\Gamma_{\alpha v}^{\beta }+\frac{2}{3}\Gamma_{\mu \gamma }^{\beta }\Gamma_{v\alpha }^{\gamma }\right)\right], \end{array}$$ (17)

where $\kappa =2/\sqrt{M_{\mathrm{Pl}}}\;$ is the gravitational coupling constant and *M*_Pl_ denotes the Planck mass $M_{\mathrm{Pl}}=\frac{\;1}{8\pi G},$ with *G* being the Newton constant.

We note that the 4-point physical gauge boson scattering amplitude (Eq. 13) is invariant under the generalized gauge transformation:

$$N_{j}\;\to N_{j}^{\prime }=N_{j}+\Delta \;\left(s_{j}-m^{2}\right),$$ (18)

where the index *j* = *s*, *t*, *u* and the coefficient Δ is an arbitrary function of kinematic variables. We find that the numerators $\left\{N_{j}\right\}$ of Eq. 13 do not manifestly obey the kinematic Jacobi identity, namely, $\sum_{j}‍N_{j}\neq 0\;$. Then, we require the gauge-transformed numerators $\left\{N_{j}^{\prime }\right\}$ to satisfy the Jacobi identity $\sum_{j}N_{j}^{\prime }=0\;$, with which we determine the coefficient Δ as follows:

$$\begin{array}{c} \begin{array}{l} \Delta =\;-\frac{i}{\;32m^{3}s_{\theta }\;}\left[\left(16m^{4}s^{-\frac{1}{2}}+8m^{2}s^{\frac{1}{2}}-3s^{\frac{3}{2}}\right)\right.\\ \left.\;-\left(16m^{4}s^{-\frac{1}{2}}-24m^{2}s^{\frac{1}{2}}-3s^{\frac{3}{2}}\right)c_{2\theta }+i16{\mathrm{ss}}_{\theta }\right]. \end{array} \end{array}$$ (19)

With this, we present the full expressions of the gauge-transformed numerators $\left\{N_{j}^{\prime }\right\}$ in Eq. S23 [39]. Thus, from Eq. 13, we can derive the following gauge-transformed new scattering amplitude:

$$\begin{array}{c} T^{\prime }\left[4A_{P}^{a}\right]=g^{2}\left(\;,\frac{\;C_{s}N_{s}^{\prime }\;}{\;s-m^{2}\;},+,\frac{\;C_{t}\;N_{t}^{\prime }\;}{\;t-m^{2}\;},+,\frac{\;C_{u}N_{u}^{\prime }\;}{\;u-m^{2}\;}\right) \end{array}.$$ (20)

We find that each $N_{j}^{\prime }$ scales as *E*^3^ under high-energy expansion, and thus, each term in the gauge boson amplitude (with numerators given by $\left\{N_{j}^{\prime }\right\}$) (Eq. 13) should scale as *E*^1^. Using the gauge-transformed numerators $\left\{N_{j}^{\prime }\right\}$, the individual terms of the amplitude (Eq. 20) have leading contribution scales as *E*^1^ instead of *E*^3^. We can verify the exact cancelation of the leading $O\left(E^{1}\right)$ contributions by summing them up into the following form:

$$T^{\prime }{\left[4A_{P}^{a}\right]}_{E^{1}}=-\frac{\;ig^{2}\sqrt{s\;}\;}{8m}\;\frac{\;\left(7+c_{2\theta }\right)\;}{s_{\theta }}\left(C_{s}+C_{t}+C_{u}\right)=0,$$ (21)

which is proportional to the Jacobi identity and vanishes identically. This cancelation happens in a similar fashion as the last column of Table 1, but the sum of all terms of last column of Table 1 gives a rather different coefficient (containing distinctive angular dependence):

$$\;T{\left[4A_{P}^{a}\right]}_{E^{1}}=-\frac{i5g^{2}\sqrt{s\;}\;}{4m}s_{\theta }\left(C_{s}+C_{t}+C_{u}\right)=0.$$ (22)

We have further verified that using the amplitude (Eq. 20) with the gauge-transformed numerators $\left\{N_{j}^{\prime }\right\}$ and making high-energy expansion, the nonzero leading contribution to the gauge boson scattering amplitude (Eq. 13) takes the same form as that of Eq. 14 at $O\left(E^{0}\right)$. This supports our conclusion that the leading nonzero gauge boson anmplitudes at $O\left(E^{0}\right)$ are universal, which are independent of the choice of the expansion parameters (such as $1/\bar{s}$ or $1/{\bar{s}}_{0}$) and independent of the basis choice of numerators ($N_{j}$ or $N_{j}^{\prime }$ as connected by the gauge transformations).

Next, we use the power counting rule (Eq. 12) to count the leading high-energy dependence of the 4-graviton scattering amplitude at tree level. The 4-graviton amplitude receives contributions from the contact diagram and the pole diagrams via (*s*, *t*, *u*) channels, as shown in the second row of Figure. We find that the leading contributions of the individual Feynman diagrams to the physical graviton scattering amplitude scale as *E*^12^. However, using the extended double-copy approach, we will uncover a series of striking energy cancelations in the 4-graviton scattering amplitudes, which make the summation of energy-dependent terms cancel all the way from $O\left(E^{12}\right)$ down to $O\left(E^{1}\right)$.

For this purpose, we extend the conventional massless double-copy method [18–20] to the case of TMYM theories. Applying the correspondence of the extended color–kinematics duality $C_{j}\to N_{j}^{\prime }\;\;$ to the gauge-transformed 4-point massive gauge boson amplitude (Eq. 20), we construct the scattering amplitude of massive gravitons with physical polarization, $M\left[h_{P}h_{P}\to h_{P}h_{P}\right]\equiv M\left[4h_{P}\right]$, as follows:

$$\begin{array}{c} M\left[4h_{P}\right]=\frac{\;\kappa^{2}\;}{16}\;\left(\frac{N_{s}^{\prime 2}}{\;s-m^{2}\;}+\frac{\;N_{t}^{\prime 2}\;}{\;t-m^{2}\;}+\frac{\;N_{u}^{\prime 2}\;}{\;u-m^{2}\;}\right), \end{array}$$ (23)

where we have made the gauge–gravity coupling conversion *g* → *κ*/4. We stress that, as a key point, the above double-copy construction must be applied directly to the full gauge boson amplitude (Eq. 20) without high-energy expansion*.* Substituting the numerators $\left(N_{s}^{\prime },N_{t}^{\prime },N_{u}^{\prime }\right)$ [39] into Eq. 23, we derive the following exact tree-level scattering amplitude of massive gravitons:

$$M\left[4h_{P}\right]=\frac{\;\kappa^{2}m^{2}\left(Q_{0}+Q_{2}c_{2\theta }+Q_{4}c_{4\theta }+Q_{6}c_{6\theta }+{\bar{Q}}_{2}s_{2\theta }+{\bar{Q}}_{4}s_{4\theta }+{\bar{Q}}_{6}s_{6\theta }\right)\;\mathrm{cs}c^{2}\;\theta \;}{4096\left(\bar{s}-1\right){\bar{s}}^{3/2}\left[\bar{s}-2-\left(\bar{s}-4\right)c_{\theta }\right]\left[\bar{s}-2+\left(\bar{s}-4\right)c_{\theta }\right]},$$ (24)

where in the numerators $\left(Q_{j},{\bar{Q}}_{j}\right)$ are polynomial functions of the dimensionless Mandelstam valiable $\bar{s}=s/m^{2}\left(={\bar{s}}_{0}+4\right)$:

$$\begin{array}{c} \begin{array}{l} \begin{array}{l} \;Q_{0}=\left(256+49088\bar{s}-68880{\bar{s}}^{2}+25220{\bar{s}}^{3}-2768{\bar{s}}^{4}\right){\bar{s}}^{\frac{1}{2}},\\ \;Q_{2}=\left(-768-45568\bar{s}+65568{\bar{s}}^{2}-19008{\bar{s}}^{3}+505{\bar{s}}^{4}\right){\bar{s}}^{\frac{1}{2}},\\ \;Q_{4}=4\left(192-176\bar{s}+20{\bar{s}}^{2}+635{\bar{s}}^{3}+58{\bar{s}}^{4}\right){\bar{s}}^{\frac{1}{2}},\\ \;Q_{6}=-\left(256+2816\bar{s}+2912{\bar{s}}^{2}+560{\bar{s}}^{3}+17{\bar{s}}^{4}\right){\bar{s}}^{\frac{1}{2}},\\ \;{\bar{Q}}_{2}=i\left(1280-256\bar{s}+21312{\bar{s}}^{2}-8960{\bar{s}}^{3}+475{\bar{s}}^{4}\right)\bar{s},\\ \;{\bar{Q}}_{4}=i4\left(320-544\bar{s}+676{\bar{s}}^{2}+272{\bar{s}}^{3}+5{\bar{s}}^{4}\right)\bar{s},\\ \;{\bar{Q}}_{6}=-i\left(1280+3584\bar{s}+1568{\bar{s}}^{2}+128{\bar{s}}^{3}+{\bar{s}}^{4}\right)\bar{s}. \end{array} \end{array} \end{array}$$ (25)

The above massive graviton scattering amplitude can be also reexpressed in terms of the Mandelstam variable *s*_0_ (=*s* − 4*m*^2^), which does not contain any mass dependence, as shown in Section S4 of the Supplementary Materials [39].

Then, we expand Eq. 24 by the high-energy expansion of 1/*s* and derive the 4-graviton scattering amplitude at the LO:

$$\;M_{0}\left[4h_{P}\right]=-\frac{\;i\kappa^{2}m\;}{\;2048\;}s^{\frac{1}{2}}\left(494c_{\theta }+19c_{3\theta }-c_{5\theta }\right)\mathrm{cs}c^{3}\theta ,$$ (26)

which has the distinctive scaling of $O\left(\mathrm{mE}\right)$. We can also make the high-energy expansion of 1/*s*_0_ (with *s*_0_ = *s* − 4*m*^2^), and the LO graviton amplitude takes the same form as Eq. 26, except the replacement $s^{1/2}\to s_{0}^{1/2}$.

From the LO graviton amplitude (Eq. 26), we can derive its *s*-partial wave amplitude *a*_0_ as follows:

Rea0=−15κ2m21024πδ3s=Oκ2m2E, (27A)

Ima0=−247κ2m224576πδ3=O(κ2mE0), (27B)

where we have added an angular cut on the scattering angle (*δ* ⩽ *θ* ⩽ *π* − *δ*) to remove the collinear divergences of the integral. We see that the above partial wave amplitudes have good high-energy behaviors and remain finite in the high-energy limit *E* → ∞. Imposing the unitarity conditions ∣Rea0∣<1/2 and ∣Ima0∣<1 [50–52], we deduce the following constraints:

$$\sqrt{s\;}>\frac{\;15\kappa^{2}m^{2}\;}{\;1024\pi \delta^{3}\;},\;m<\frac{\;49152\pi \delta^{3}\;}{247\kappa^{2}},$$ (28)

which can be readily obeyed. This shows that the 3D TMG exhibits good UV behavior, unlike the conventional 3D Fierz–Pauli type of massive gravity models.

We note that the leading individual terms of the numerators $\left(N_{j},N_{j}^{\prime }\right)$ scale as (*E*^5^, *E*^3^), respectively [39], where the gauge transformation (Eq. 18) causes the energy cancelations of *E*^5^ → *E*^3^ in each new numerator $N_{j}^{\prime }$. This has an important impact on the energy dependence of the double-copied graviton amplitude (Eq. 23). Namely, in each channel, the amplitude $N_{j}^{\prime 2}/\left(s_{j}-m^{2}\right)\;$ contains leading energy dependence behaving as *E*^4^, rather than *E*^8^ from $N_{j}^{2}/\left(s_{j}-m^{2}\right)$. In comparison with the leading energy dependence of each individual contribution of the tree-level 4-graviton amplitude that scales as *E*^12^ by the direct power counting of individual Feynman diagrams, our double-copy construction (Eq. 23) demonstrates that, in each channel, the graviton scattering amplitude could have the leading energy dependence of *E*^4^ at most. Hence, the double-copy construction guarantees a series of large-energy cancelations in the original 4-graviton scattering amplitude, *E*^12^ → *E*^4^, which brings the leading *E* dependence down by a large power factor of *E*^8^ = *E*^4 × 2^.

In fact, we further discover a series of striking energy cancelations of *E*^4^ → *E*^1^ in the full graviton scattering amplitude (Eq. 23), which rely on the sum of all 3 kinematic channels*.* We summarize these exact *E* cancelations in Table 2. We note that an *S*-matrix element S with E external states and *L* loops has mass–dimension $D_{S}=3-E/2\;$ in the 3D space–time [39]. Thus, the 4-point graviton scattering amplitude $M\left[4h_{P}\right]$ in 3D has mass–dimension of 1 and contains the gravity coupling *κ*^2^ of mass–dimension of −1. Hence, we can express the graviton scattering amplitude $M\left[4h_{P}\right]=\kappa^{2}\bar{M}\left[4h_{P}\right]$ , where $\bar{M}\left[4h_{P}\right]$ has mass–dimension 2 and is determined by the 2 dimensionful parameters (*E*, *m*). Thus, we can deduce its scaling behavior $\bar{M}\left[4h_{P}\right]\propto m^{n_{1}}E^{n_{2}}$ with *n*_1_ + *n*_2_ = 2 , under the high-energy expansion. For the energy terms of *E*^*n*_2_^ with *n*_2_ = 4, 3, 2, we deduce its corresponding mass–power factor *n*_1_ = −2, −1, 0, respectively. This means that, in the massless limit *m* → 0 , the physical graviton amplitude $\bar{M}\left[4h_{P}\right]$ would go to infinity (for *n*_2_ ⩾ 3) or would remain constant (for *n*_2_ = 2). However, we observe that, in the massless limit *m* → 0 , the 3D graviton field becomes unphysical and has no physical degrees of freedom [47–49]. Hence, the scattering amplitude $\bar{M}\left[4h_{P}\right]$ should vanish because the physical graviton *h*_P_ no longer exists in this limit. This means that the *m*^*n*_1_^*E*^*n*_2_^ terms with *n*_1_ = −2, −1, 0 should vanish and the physical scattering amplitude $\bar{M}\left[4h_{P}\right]$ has to start with the leading behavior of *m*^1^*E*^1^, just as the behavior shown in Eq. 26. This is why the energy cancelations should hold at each order of (*E*^4^, *E*^3^, *E*^2^), in accord with what we have discovered in Table 2 by the explicit analysis of the energy structure of the massive graviton amplitude (Eq. 24).

**Table 2.** Exact energy cancelations at each order of (*E*^4^, *E*^3^, *E*^2^) in our double-copied 4-graviton scattering amplitude (Eq. 23). A common overall factor (*κ*^2^*m*^2^/2048) in each entry is not displayed for simplicity.

Amplitude	$\times {\bar{s}}^{2}$	$\times {\bar{s}}^{3/2}$	$\times \bar{s}$
$M_{s}$	$-\frac{\;99+28c_{2\theta }+c_{4\theta }\;}{1-c_{2\theta }}$	−i14(15*c_θ_* + *c*_3*θ*_) csc *θ*	$\frac{\;2\left(321-214c_{2\theta }-43c_{4\theta }\right)\;}{1-c_{2\theta }}$
$M_{t}$	$\frac{\;99+28c_{2\theta }+c_{4\theta }\;}{4\left(1-c_{\theta }\right)}$	i(102 + 105*c_θ_* + 70*c*_2*θ*_ + 7*c*_3*θ*_ + 4*c*_4*θ*_) csc *θ*	$-\frac{\;321+559c_{\theta }-214c_{2\theta }-210c_{3\theta }-43c_{4\theta }-29c_{5\theta }}{1-c_{2\theta }}$
$M_{u}$	$\frac{\;99+28c_{2\theta }+c_{4\theta }\;}{4\left(1+c_{\theta }\right)}$	i(−102 + 105*c_θ_* − 70*c*_2*θ*_ + 7*c*_3*θ*_ − 4*c*_4*θ*_) csc *θ*	$-\frac{\;321-559c_{\theta }-214c_{2\theta }+210c_{3\theta }-43c_{4\theta }+29c_{5\theta }}{1-c_{2\theta }}$
Sum	0	0	0

Finally, for possible applications to the condensed matter system and other low-energy studies, we analyze the nonrelativistic limit and make the low-energy expansion of the double-copied 4-graviton scattering amplitude (Eq. 24). Thus, we derive the following LO and NLO scattering amplitudes of massive gravitons under the low-energy expansion:

$$\;M_{0}\left[4h_{P}\right]=\frac{\;\kappa^{2}m^{2}\;}{\;16s_{\theta }^{2}\;}\left(-73+73c_{2\theta }+i7s_{2\theta }\right)e^{i4\theta },$$ (29A)

$$\;\delta M\left[4h_{P}\right]=\frac{\;\kappa^{2}s\;}{\;64\;s_{\theta }^{2}\;}\left(55-47c_{2\theta }-i17s_{2\theta }\right)e^{i4\theta },$$ (29B)

where *s* ≪ *m*^2^. It shows that, under nonrelativistic expansion, the LO graviton amplitude $M_{0}\left[4h_{P}\right]$ scales as *E*^0^*m*^2^ and the NLO graviton amplitude $\delta M\left[4h_{P}\right]$ behaves as *E*^2^*m*^0^.

## Conclusions and Discussions

Studying the mechanism of topological mass generations and its impact on the structure the massive gauge boson/graviton scattering amplitudes in the 3D CS theories is important for applying the modern quantum field theories to particle physics and condensed matter physics [1–4]. In this Letter, we systematically studied the high-energy behaviors of the gauge boson/graviton scattering amplitudes in the TMYM theory and the TMG theory [1,2]. We found that making the high-energy expansion uncovers large-energy cancelations *E*^4^ → *E*^4 − *N*^ for each *N*-point massive gauge boson scattering amplitude. These energy cancelations are ensured by the TET identity (Eq. 9A and B) as we newly proposed in the “Formulation of TET” section. This is highly nontrivial because naively taking the massless limit would cause the (physical, longitudinal) polarization vectors in Eq. 3A and B to diverge, $(\;\epsilon_{P}^{\mu },\epsilon_{L}^{\mu }\;)\to \infty$, and thus make the physical state of the topologically massive gauge boson $A_{P}^{a}$ ill-defined. The nontrivial and consistent approach is to take the high-energy expansion for a fixed nonzero gauge boson mass *m* ≠ 0 and prove the large-energy cancelations by using the TET identity (Eq. 9A and B), as we demonstrated in the section of “Formulation of the TET”. Moreover, we further extended the conventional massless double-copy approach to the present massive TMYM and TMG theories. We constructed the massive 4-graviton scattering amplitude and uncovered its structure as in Eqs. 23 to 26 and Table 2. A key point is that the double-copy construction must be applied to the exact gauge boson amplitude (Eq. 20) without high-energy expansion. From these, we discovered a series of strikingly large energy cancelations in the 4-point massive graviton scattering amplitude at tree level:

$$O\left(E^{12}\right)\to O\left(E^{1}\right),$$ (30)

for the 3D TMG theory. Our analysis has newly established a striking correspondence between the 2 types of distinctive energy cancelations of 4-point massive scattering amplitudes: *E*^4^ → *E*^0^ in the TMYM theory and *E*^12^ → *E*^1^ in the TMG theory. In Eq. 30, the exact energy cancelations in the 4-graviton scattering amplitude by a large power of *E*^11^ are even much more severe than the energy cancelations *E*^10^ → *E*^2^ in the massive 4-longitudinal KK graviton scattering amplitudes of the compactified 5D gravity theory as found by explicit calculations [53–55] and by the KK double-copy construction [16,17]. Our discovery of the striking energy cancelations of *E*^12^ → *E*^1^ newly demonstrates that the massive graviton scattering amplitudes in the 3D TMG theory have much better UV behavior than the naive expectation based on the conventional power counting of Feynman diagrams. This also encourages us to further establish the renormalizability of the TMG theory by extending our massive double-copy approach up to loop levels. For the possible applications to the condensed matter system and other low-energy studies, we further presented the nonrelativistic scattering amplitudes of the massive gauge bosons in Eq. 16A and B and of the massive gravitons in Eq. 29A and B. A substantial extension of the main content of this Letter is presented in our companion long paper [56] (where the nonrelativistic scattering amplitudes are not shown).

## Data Availability

All data needed for the current work are presented in this paper and the Supplementary Materials. Additional data related to this paper may be requested from the authors.

## Supplementary Materials

Supplementary 1 Section S1. Kinematics of 4-particle scattering. Section S2. Polarization states and Feynman rules in 3D CS gauge theories. Section S3. Power counting method for 3D CS theories. Section S4. Scattering amplitudes for the TMYM and TMG theories. Table S1. Energy cancelations for amplitude $T\left[4A_{P}^{a}\right]=T_{c}+T_{s}+T_{t}+T_{u}T\left[4A_{P}^{a}\right]=T_{c}+T_{s}+T_{t}+T_{u}$ in the 3D TMYM theory, where the contribution of the contact channel is decomposed into 3 sub-amplitudes according to the color factors, $T_{c}=T_{\mathrm{cs}}+T_{\mathrm{ct}}+T_{\mathrm{cu}}$. Table S2. Exact energy cancelations at each order of (*E*^4^, *E*^3^, *E*^2^) in our double-copied 4-graviton scattering amplitude (22). Reference [57].
