# Supplementary material for: Topological Equivalence Theorem and Double-Copy for Chern–Simons Scattering Amplitudes
Source: Research (Wash D C). 2023 Mar 17;6:0072. doi: 10.34133/research.0072 (PMC10766434; doi:10.34133/research.0072)
Supplement: Supplementary 1 — Section S1. Kinematics of 4-particle scattering. Section S2. Polarization states and Feynman rules in 3D CS gauge theories. Section S3. Power counting method for 3D CS theories. Section S4. Scattering amplitudes for the TMYM and TMG theories. Table S1. Energy cancelations for amplitude T4APa=Tc+Ts+Tt+TuT4APa=Tc+Ts+Tt+Tu in the 3D TMYM theory, where the contribution of the contact channel is decomposed into 3 sub-amplitudes according to the color factors, Tc=Tcs+Tct+Tcu. Table S2. Exact energy cancelations at each order of (E4, E3, E2) in our double-copied 4-graviton scattering amplitude (22). Reference [57]. [file research.0072.f1.pdf]

Topological Equivalence Theorem and Double-Copy for Chern-Simons Scattering Amplitudes

— Supplementary Material —

Yan-Feng Hang [1] , Hong-Jian He [1,2,3] , and Cong Shen [1,4]

[1] Tsung-Dao Lee Institute & School of Physics and Astronomy,
Key Laboratory for Particle Astrophysics and Cosmology (MOE),
Shanghai Key Laboratory for Particle Physics and Cosmology,
Shanghai Jiao Tong University, Shanghai, China

[2] Institute of Modern Physics & Physics Department, Tsinghua University, Beijing, China

[3] Center for High Energy Physics, Peking University, Beijing, China

[4] Fields and Strings Laboratory, Institute of Physics,
Ecole Polytechnique Federale de Lausanne, Switzerland

(yfhang@sjtu.edu.cn, hjhe@sjtu.edu.cn, cong.shen@epfl.ch)

This Supplementary Material provides in detail the relevant formulas for the analyses of scattering amplitudes in the 3d Chern-Simons (CS) gauge theory and their double-copy for the 3d Topologically Massive Gravity (TMG) theory. In Section 1, we define the kinematics for the four-point scattering in the 3d spacetime. In section 2, we derive polarization vectors for the physical gauge boson states, and present the Feynman rules of the 3d Topologically Massive Yang-Mills (TMYM) theory. In Section 3, we present the generalized power counting method for the scattering amplitudes of the 3d CS gauge theories and the 3d TMG theory. In Section 4, we present the complete formulas for the kinematic numerators of the four-point gauge boson scattering amplitudes at tree level (before and after the generalized gauge transformations). Then, we present the exact double-copied four-graviton amplitude in terms of the Mandelstam variable $s_0\,(=s-4m^2)$ and its expanded formulas under the high energy expansion of m^2/s_0 .

1 Kinematics of Four-Particle Scattering

The 3d Minkowski metric tensor and rank-3 Levi-Civita tensor are defined as follows:

$$\eta^{\mu\nu}=\eta_{\mu\nu}=\begin{pmatrix}-1 & & \\ & 1 & \\ & & 1\end{pmatrix}, \qquad \varepsilon^{012}=-\varepsilon_{012}=1. \tag{S1}$$

The external momenta for the $2\!\rightarrow\!2$ elastic scattering process in the center-of-mass frame are given by

$$\begin{aligned} p_1^\mu &= E(1,0,\beta), & p_2^\mu &= E(1,0,-\beta), \\ p_3^\mu &= E(1,\beta s_\theta,\beta c_\theta), & p_4^\mu &= E(1,-\beta s_\theta,-\beta c_\theta), \end{aligned} \tag{S2}$$

where the velocity $\beta=\sqrt{1-m^2/E^2}$ and $(s_\theta,c_\theta)=(\sin\theta,\,\cos\theta)$ with θ being the scattering angle. Hence, we can use the momenta (S2) to define the three Mandelstam variables (s,t,u) as follows:

$$s=-\,(p_1+p_2)^2=4E^2, \quad t=-\,(p_1-p_4)^2=-\frac{s}{2}\beta^2(1+c_\theta), \quad u=-\,(p_1-p_3)^2=-\frac{s}{2}\beta^2(1-c_\theta). \tag{S3}$$

In the present analysis, we use the on-shell relation $E^2 = E^2\beta^2 + m^2$ to define a new set of mass-independent Mandelstam variables (s_0, t_0, u_0) as follows [1]:

$$s_0 \,=\, 4E^2\beta^2, \qquad t_0 \,=\, -\tfrac{1}{2}s_0(1{+}c_\theta), \qquad u_0 \,=\, -\tfrac{1}{2}s_0(1{-}c_\theta), \tag{S4}$$

where $(s,\, s_0)$ are connected by $s_0 = s - 4m^2$. Furthermore, the summations of (s, t, u) and (s_0, t_0, u_0) satisfy the following relations:

$$s + t + u = 4m^2, \qquad s_0 + t_0 + u_0 = 0\,. \tag{S5}$$

2 Polarization States and Feynman Rules in 3d CS Gauge Theories

The little groups for massless and massive particles in 3d spacetime are $\mathbb{Z}_2 \otimes \mathbb{R}$ and SO(2), respectively [2]. The 3d Poincaré group is ISO(2,1), which contains the proper Lorentz group SO(2,1) and the spacetime translations [2][3]. The 3d Poincaré algebra is characterized by two Casimir operators $(P^2,\, W) = (P_\mu P^\mu,\, P_\mu J^\mu)$, where W is the Pauli-Lubanski pseudo-scalar and the angular momentum J^μ can be generally expressed as follows [4]:

$$J^\mu \,=\, -\mathrm{i}\varepsilon^{\mu\nu\alpha}p_\nu \frac{\partial}{\partial p_\alpha} - \mathfrak{s}\frac{p^\mu{+}\eta^\mu m}{p{\cdot}\eta - m}\,, \tag{S6}$$

with $\eta^\mu = (1,0,0)$. Thus, the Pauli-Lubanski pseudo-scalar is given by $W = P_\mu J^\mu = -\mathfrak{s}m$ in the rest frame. Hence the spin $\mathfrak{s}$ is also a pseudo-scalar and takes the values $\mathfrak{s} = \pm 1$ for gauge fields A^a_μ. The polarization with either $\mathfrak{s} = +1$ or $\mathfrak{s} = -1$ is physically equivalent.

In the rest frame, we can solve the equation of motion (2) in the main text for the momentum $\bar{p}^\mu = (m,\, 0,\, 0)$:

$$\epsilon^\mu(\bar{p}) = \tfrac{1}{\sqrt{2}}(0,\, 1,\, -\mathrm{i}\mathfrak{s})\,. \tag{S7}$$

We note that in the rest frame the above gauge boson polarization vector has zero time-component and its two possible forms are not independent due to the relation $\epsilon_2^\mu = \mathrm{i}\mathfrak{s}\epsilon_1^\mu$. We can further choose the orthonormal basis $e_1^j = (1,0)$ and $e_2^j = (0,1)$ in a plane, and define a polarization basis:

$$e_\pm^j \,=\, \tfrac{1}{\sqrt{2}}(e_1^j \pm \mathrm{i}e_2^j) \,=\, \tfrac{1}{\sqrt{2}}(1,\, \pm\mathrm{i})\,. \tag{S8}$$

Thus, in the rest frame we can express the spatial components of the polarization vector $\epsilon^\mu(\bar{p})$ under the basis $\{e_\pm^j\}$:

$$\epsilon^j(\bar{p}) \,=\, \epsilon_+ e_+^j + \epsilon_- e_-^j\,, \tag{S9}$$

where the coefficients $(\epsilon_+,\, \epsilon_-)$ satisfy $(\epsilon_+,\, \epsilon_-) = (0,\, 1)$ for $\mathfrak{s} = +1$ and $(\epsilon_+,\, \epsilon_-) = (1,\, 0)$ for $\mathfrak{s} = -1$ [5]. So, as we expect, for the 3d Chern-Simons gauge theory, the $\mathfrak{s} = +1$ case (or, $\mathfrak{s} = -1$ case) only allows one physical polarization state ϵ_- (or, ϵ_+) of the gauge boson.

Then, we can make a Lorentz transformation to boost the polarization vector (S7) in the rest frame to the following polarization vector for a general momentum $p^\mu = E(1,\, \beta s_\theta,\, \beta c_\theta)$:

$$\epsilon_{\mathrm{P}}^\mu(p) \,=\, \frac{1}{\sqrt{2}}\left(\frac{\mathrm{i}p_1{+}\mathfrak{s}p_2}{m}\,,\ \mathrm{i}{+}\frac{p_1(\mathrm{i}p_1{+}\mathfrak{s}p_2)}{m(m{-}p_0)}\,,\ \mathfrak{s}{+}\frac{p_2(\mathrm{i}p_1{+}\mathfrak{s}p_2)}{m(m{-}p_0)}\right), \tag{S10}$$

where $\epsilon_{\mathrm{P}+}^\mu = -(\epsilon_{\mathrm{P}-}^\mu)^*$. Thus, we substitute the momenta (S2) into Eq.(S10), and derive the following explicit form of the polarization vectors:

$$\epsilon_1^\mu \,=\, \frac{\mathfrak{s}}{\sqrt{2}}(\bar{E}\beta,\, \mathrm{i}\mathfrak{s},\, \bar{E}), \qquad \epsilon_2^\mu \,=\, -\frac{\mathfrak{s}}{\sqrt{2}}(\bar{E}\beta,\, -\mathrm{i}\mathfrak{s},\, -\bar{E}),$$

$$\epsilon_3^\mu = \frac{\mathfrak{s}e^{\mathrm{i}\mathfrak{s}\theta}}{\sqrt{2}}\big(\bar{E}\beta,\, \bar{E}s_\theta + \mathrm{i}\mathfrak{s}c_\theta,\, \bar{E}c_\theta - \mathrm{i}\mathfrak{s}s_\theta\big), \quad \epsilon_4^\mu = -\frac{\mathfrak{s}\,e^{\mathrm{i}\mathfrak{s}\theta}}{\sqrt{2}}\big(\bar{E}\beta,\, -\bar{E}s_\theta - \mathrm{i}\mathfrak{s}c_\theta,\, -\bar{E}c_\theta + \mathrm{i}\mathfrak{s}s_\theta\big), \tag{S11}$$

where we denote a dimensionless energy factor $\bar{E} = E/m$.

Next, we derive the Feynman rules for the Chern-Simons (CS) gauge theories. We consider the non-Abelian case of the Topologically Massive Yang-Mills (TMYM) theory, and the Abelian case corresponds to a special case by setting all the color indices equal one and the group structure constant $C^{abc} = 0$. Thus, the gauge boson propagator for $\mathfrak{s} = \widetilde{m}/m = +1$ is given by

$$\mathcal{D}_{\mu\nu}^{ab}(p) = -\mathrm{i}\delta^{ab}\left[\frac{1}{p^2+m^2}\left(\eta_{\mu\nu} - \frac{p_\mu p_\nu}{p^2} - \frac{\mathrm{i}m\varepsilon_{\mu\nu\rho}p^\rho}{p^2}\right) + \xi\frac{p_\mu p_\nu}{p^4}\right]. \tag{S12}$$

The massless pole of the propagator (S12) is unphysical because taking $p^2 = 0$ with $m \neq 0$ in the original equation of motion [7] leads to $\mathfrak{s}m\varepsilon^{\mu\rho\nu}p_\rho\epsilon_\nu = 0$ and thus gives the solution of the polarization vector, $\epsilon^\mu \propto f(p)p^\mu$, which can be eliminated by the freedom of gauge transformations [6].

Then, we derive the Feynman rules of cubic and quartic gauge boson vertices as follows:

$$V_{\mu\nu\alpha}^{abc} = gC^{abc}[\,\eta_{\mu\nu}(p_1 - p_2)_\alpha + \eta_{\nu\alpha}(p_2 - p_3)_\mu + \eta_{\alpha\mu}(p_3 - p_1)_\nu + \mathrm{i}m\varepsilon_{\mu\nu\alpha}\,], \tag{S13a}$$

$$V_{\mu\nu\alpha\beta}^{abcd} = -\mathrm{i}g^2\begin{bmatrix} C^{abe}C^{cde}(\eta_{\mu\alpha}\eta_{\nu\beta} - \eta_{\mu\beta}\eta_{\nu\alpha}) \\ + C^{ace}C^{dbe}(\eta_{\mu\beta}\eta_{\nu\alpha} - \eta_{\mu\nu}\eta_{\alpha\beta}) \\ + C^{ade}C^{bce}(\eta_{\mu\nu}\eta_{\alpha\beta} - \eta_{\mu\alpha}\eta_{\nu\beta}) \end{bmatrix}, \tag{S13b}$$

where the structure constant appears in the commutator $[T^a, T^b] = \mathrm{i}C^{abc}T^c$, with T^a denoting the generator of the gauge group $\mathrm{SU}(N)$.

3 Power Counting Method for 3d Chern-Simons Theories

Consider a scattering S-matrix element $\mathbb{S}$ having $\mathcal{E}$ external states and L loops ($L \geqslant 0$). Thus, the amplitude $\mathbb{S}$ has a mass-dimension [7]:

$$D_{\mathbb{S}} = 3 - \tfrac{1}{2}\mathcal{E}, \tag{S14}$$

where the number of external states $\mathcal{E} = \mathcal{E}_B + \mathcal{E}_F$ with $\mathcal{E}_B$ and $\mathcal{E}_F$ being the numbers of external bosonic and fermionic states, respectively. We denote the number of vertices of type-j as $\mathcal{V}_j$, where each vertex $\mathcal{V}_j$ includes d_j derivatives, b_j bosonic lines, and f_j fermionic lines. Then, the total mass-dimension of the energy-independent coupling constant in the amplitude $\mathbb{S}$ is given by

$$D_C = \sum_j \mathcal{V}_j\big(3 - d_j - \tfrac{1}{2}b_j - f_j\big). \tag{S15}$$

For each Feynman diagram contributing to the amplitude $\mathbb{S}$, we denote the number of the internal lines as $I = I_B + I_F$ with I_B (I_F) being the number of the internal bosonic (fermionic) lines. Thus, we have the following general relations:

$$L = 1 + I - \mathcal{V}, \qquad \sum_j \mathcal{V}_j b_j = 2I_B + \mathcal{E}_B, \qquad \sum_j \mathcal{V}_j f_j = 2I_F + \mathcal{E}_F, \tag{S16}$$

where $\mathcal{V} = \sum_j \mathcal{V}_j$ is the total number of vertices in a given Feynman diagram. Hence, from Eqs.(S14)-(S16), we derive the leading energy-power dependence of the amplitude $\mathbb{S}$ as follows:

$$D_E = D_{\mathbb{S}} - D_C = 2(1 - \mathcal{V}) + L + \sum_j \mathcal{V}_j\big(d_j + \tfrac{1}{2}f_j\big). \tag{S17}$$

Then, we note that the following relations must be obeyed:

$$\sum_j \mathcal{V}_j d_j = \mathcal{V}_d\,, \quad \sum_j \mathcal{V}_j f_j = 2\mathcal{V}_F\,, \quad \mathcal{V}=\sum_j \mathcal{V}_j = \mathcal{V}_3+\mathcal{V}_4\,, \quad \mathcal{V}_3=\mathcal{V}_d+\mathcal{V}_F+\overline{\mathcal{V}}_3\,, \tag{S18}$$

where $\mathcal{V}_d$ denotes the number of all cubic vertices including one partial derivative and $\overline{\mathcal{V}}_3$ denotes the number of cubic vertices without partial derivative [1]. With these, we can derive the following power counting rule on the leading energy dependence:

$$D_E \;=\; (4-\mathcal{E}-\overline{\mathcal{V}}_3)+(\mathcal{E}_{A_\mathrm{P}}-\mathcal{E}_v)-L\,, \tag{S19}$$

where $\mathcal{E}_{A_\mathrm{P}}$ denotes the number of external physical gauge bosons $A^a_\mathrm{P}\,(=\epsilon^\mu_\mathrm{P} A^a_\mu)$ and $\mathcal{E}_v$ represents the number of external gauge boson states contracted with the factor v^μ.

For the topologically massive gravity (TMG) theory, we note that the leading graviton self-interaction vertex comes from the CS term which always contains 3 partial derivatives. Thus, for a given graviton vertex of this kind we have $d_j=3$ and $f_j=0$ in Eq.(S17), which lead to $\sum_j \mathcal{V}_j d_j = 3\mathcal{V}_{d3}$ and $\mathcal{V}=\mathcal{V}_{d3}$ in such leading diagrams, where $\mathcal{V}_{d3}$ is the number of vertices including 3 partial derivatives. Hence, the leading energy contribution to the pure graviton scattering amplitude in 3d spacetime is given by the Feynman diagrams including the CS graviton vertices with 3 derivatives, and thus is determined as follows:

$$D_E \;=\; 2\mathcal{E}_{h_\mathrm{P}}+(\mathcal{V}_{d3}+L+2)\,, \tag{S20}$$

where $\mathcal{E}_{h_\mathrm{P}}$ denotes the number of external physical graviton states $h_\mathrm{P}\,(=\epsilon^{\mu\nu}_\mathrm{P} h_{\mu\nu})$ and $\mathcal{V}_{d3}$ represents the number of vertices including 3 partial derivatives. For the tree-level diagrams, we have $L=0$ and $\mathcal{V}_{d3}=\mathcal{E}_{h_\mathrm{P}}-2$. Hence, we can further express the leading energy-power dependence (S20) as follows:

$$D^0_E \;=\; 3\mathcal{E}_{h_\mathrm{P}}\,. \tag{S21}$$

4 Scattering Amplitudes for the TMYM and TMG Theories

For the theories of TMYM and TMG theories, we consider the scattering processes $A^a_\mathrm{P}A^b_\mathrm{P}\!\to\! A^c_\mathrm{P}A^d_\mathrm{P}$ ($A^a_\mathrm{T}A^b_\mathrm{T}\to A^c_\mathrm{T}A^d_\mathrm{T}$) and $h_\mathrm{P}h_\mathrm{P}\to h_\mathrm{P}h_\mathrm{P}$. The relevant Feynman diagrams are presented in Fig. 1 of the main text.

We systematically derive the kinematic numerators ($\mathcal{N}_s$, $\mathcal{N}_t$, $\mathcal{N}_u$) in the four-gauge boson scattering amplitude (13) (main text) which takes the following form:

$$\mathcal{N}_s = \frac{4m^2-s}{16m^3 s^{\frac12}}\Big[4m s^{\frac12}(5m^2+4s)c_\theta+\mathrm{i}(4m^4+29m^2 s+3s^2)s_\theta\Big], \tag{S22a}$$

$$\mathcal{N}_t = -\frac{c_{\theta/2}}{16m^3}\Big(s^{\frac12}+\mathrm{i}2m\tan\tfrac{\theta}{2}\Big)^2\Big\{4m[13m^2-3s+(8m^2-s)c_\theta]c_{\theta/2}+\mathrm{i}s^{\frac12}[22m^2-3s+(20m^2-3s)c_\theta]s_{\theta/2}\Big\}, \tag{S22b}$$

$$\mathcal{N}_u = \frac{s_{\theta/2}}{16m^3}\Big(s^{\frac12}-\mathrm{i}2m\cot\tfrac{\theta}{2}\Big)^2\Big\{4m[13m^2-3s-(8m^2-s)c_\theta]s_{\theta/2}-\mathrm{i}s^{\frac12}[22m^2-3s-(20m^2-3s)c_\theta]c_{\theta/2}\Big\}, \tag{S22c}$$

where we denote $(c_{\theta/2},\, s_{\theta/2})=(\sin\frac{\theta}{2},\, \cos\frac{\theta}{2})$. By making the gauge transformation (18) (main text) on the kinematic numerators ($\mathcal{N}_s$, $\mathcal{N}_t$, $\mathcal{N}_u$), we further derive a new set of kinematic numerators ($\mathcal{N}'_s$, $\mathcal{N}'_t$, $\mathcal{N}'_u$) which obey the Jabcobi identity and take following form:

$$\mathcal{N}'_s = \frac{\mathrm{i}\csc\theta}{8m s^{\frac12}}\big[8m^4+26m^2 s-7s^2-(8m^4+18m^2 s+s^2)c_{2\theta}-\mathrm{i}m s^{\frac12}(20m^2+7s)s_{2\theta}\big], \tag{S23a}$$

Amplitude	$\times\bar{s}_0^2$	$\times\bar{s}_0^{3/2}$	$\times\bar{s}_0$	$\times\bar{s}_0^{1/2}$
$\mathcal{T}_{cs}$	$8s_\theta\,\mathcal{C}_s$	$\mathrm{i}32s_\theta\,\mathcal{C}_s$	$64c_\theta\,\mathcal{C}_s$	$\mathrm{i}64s_\theta\,\mathcal{C}_s$
$\mathcal{T}_{ct}$	$-(5+4c_\theta-c_{2\theta})\,\mathcal{C}_t$	$-\mathrm{i}8(2s_\theta-s_{2\theta})\,\mathcal{C}_t$	$-32(c_\theta-c_{2\theta})\,\mathcal{C}_t$	$-\mathrm{i}16(2s_\theta-5s_{2\theta})\,\mathcal{C}_t$
$\mathcal{T}_{cu}$	$(5-4c_\theta-c_{2\theta})\,\mathcal{C}_u$	$-\mathrm{i}8(2s_\theta+s_{2\theta})\,\mathcal{C}_u$	$-32(c_\theta+c_{2\theta})\,\mathcal{C}_u$	$-\mathrm{i}16(2s_\theta+5s_{2\theta})\,\mathcal{C}_u$
$\mathcal{T}_s$	$-8s_\theta\,\mathcal{C}_s$	$-\mathrm{i}56s_\theta\,\mathcal{C}_s$	$-192c_\theta\,\mathcal{C}_s$	$-\mathrm{i}368s_\theta\,\mathcal{C}_s$
$\mathcal{T}_t$	$(5+4c_\theta-c_{2\theta})\,\mathcal{C}_t$	$-\mathrm{i}8(s_\theta+s_{2\theta})\,\mathcal{C}_t$	$-32(3c_\theta+c_{2\theta})\,\mathcal{C}_t$	$-\mathrm{i}16(17s_\theta+5s_{2\theta})\,\mathcal{C}_t$
$\mathcal{T}_u$	$-(5-4c_\theta-c_{2\theta})\,\mathcal{C}_u$	$-\mathrm{i}8(s_\theta-s_{2\theta})\,\mathcal{C}_u$	$-32(3c_\theta-c_{2\theta})\,\mathcal{C}_u$	$-\mathrm{i}16(17s_\theta-5s_{2\theta})\,\mathcal{C}_u$
Sum	0	0	0	0

Table S1: Energy cancellations for amplitude $\mathcal{T}[4A_{\rm P}^a]=\mathcal{T}_c+\mathcal{T}_s+\mathcal{T}_t+\mathcal{T}_u$ in the 3d TMYM theory, where the contribution of the contact channel is decomposed into three sub-amplitudes according to the color factors, $\mathcal{T}_c=\mathcal{T}_{cs}+\mathcal{T}_{ct}+\mathcal{T}_{cu}$. The energy factors are $\bar{s}_0=s_0/m^2=4\bar{E}^2\beta^2$ and $\bar{E}=E/m$, whereas for the angular dependence the notations are $(s_{n\theta},\,c_{n\theta})=(\sin n\theta,\,\cos n\theta)$. A common overall factor $(g^2/128)$ in each amplitude is not displayed for simplicity.

$$\mathcal{N}_t' = -\frac{\mathrm{i}\csc\theta}{32m\,s^{\frac{1}{2}}}\Big[(16m^4+52m^2s-14s^2)+\big(16m^4+104m^2s-15s^2\big)c_\theta-2\big(8m^4+18m^2s+s^2\big)c_{2\theta} + \big(16m^4+24m^2s+s^2\big)c_{3\theta}+\mathrm{i}ms^{\frac{1}{2}}(176m^2+20s)s_\theta-\mathrm{i}ms^{\frac{1}{2}}(40m^2+14s)s_{2\theta}-\mathrm{i}ms^{\frac{1}{2}}(32m^2+8s)s_{3\theta}\Big], \tag{S23b}$$

$$\mathcal{N}_u' = -\frac{\mathrm{i}\csc\theta}{32m\,s^{\frac{1}{2}}}\Big[(16m^4+52m^2s-14s^2)-\big(16m^4+104m^2s-15s^2\big)c_\theta-2\big(8m^4+18m^2s+s^2\big)c_{2\theta} + \big(16m^4+24m^2s+s^2\big)c_{3\theta}-\mathrm{i}ms^{\frac{1}{2}}(176m^2-20s)s_\theta-\mathrm{i}ms^{\frac{1}{2}}(40m^2+14s)s_{2\theta}+\mathrm{i}ms^{\frac{1}{2}}(32m^2+8s)s_{3\theta}\Big], \tag{S23c}$$

where we have defined the notations $(s_{n\theta},\,c_{n\theta})=(\sin n\theta,\,\cos n\theta)$.

In the main text, we have expanded the four-point gauge boson scattering amplitudes (13) using the above numerators (S22) under the high energy expansion of $1/\bar{s}$. We explicitly demonstrated the exact energy cancellations under the expansion of $1/\bar{s}$ at each order of E^n $(n=4,3,2,1)$, which are summarized in Table 1. We have also demonstrated the energy cancellations under the expansion of $1/\bar{s}_0$ at each order of E^n $(n=4,3,2,1)$, which we summarize in the above Table S1. After all these energy cancellations, we have presented the leading nonzero gauge boson amplitudes in Eq.(15) under the under the $1/\bar{s}_0$ expansion. We find that the amplitude $\mathcal{T}_0'[4A_{\rm P}^a]$ in Eq.(15) differs from the amplitude $\mathcal{T}_0[4A_{\rm P}^a]$ in Eq.(14) by an amount propotional to the Jacobi identity, so they are equivalent. We also find that the amplitude $\mathcal{T}_0[4A_{\rm T}^a]$ in Eq.(14) (under $1/\bar{s}$ expansion) and the amplitude $\mathcal{T}_0'[4A_{\rm T}^a]$ in Eq.(15) (under $1/\bar{s}_0$ expansion) are simply equal. Hence, the leading nonzero amplitudes of $\mathcal{O}(E^0)$ are universal and independent of the high-energy expansion parameters (either $1/\bar{s}$ or $1/\bar{s}_0$). Using the gauge-transformed numerators (S23) and making high energy expansion, we have further analyzed the gauge boson scattering amplitude (20) and find that its leading individual terms scale as E^1. From these, we have demonstrated the remaining energy cancellation of all the $\mathcal{O}(E^1)$ terms as shown in Eq.(21) of the main text, whereas the $\mathcal{O}(E^1)$ cancellation of the gauge boson amplitude (13) with numerators (S22) works in a similar fashion but with different coefficient as shown in Eq.(22) of the main text.

Amplitude	$\times\bar{s}_0^2$	$\times\bar{s}_0^{3/2}$	$\times\bar{s}_0$
$\mathcal{M}_s$	$-\frac{99+28c_{2\theta}+c_{4\theta}}{1-c_{2\theta}}$	$-\mathrm{i}\,14(15c_\theta+c_{3\theta})\csc\theta$	$-\frac{2(75+326c_{2\theta}+47c_{4\theta})}{1-c_{2\theta}}$
$\mathcal{M}_t$	$\frac{99+28c_{2\theta}+c_{4\theta}}{4(1-c_\theta)}$	$\mathrm{i}\,(102+105c_\theta+70c_{2\theta}+7c_{3\theta}+4c_{4\theta})\csc\theta$	$\frac{75-107c_\theta+326c_{2\theta}+268c_{3\theta}+47c_{4\theta}+31c_{5\theta}}{1-c_{2\theta}}$
$\mathcal{M}_u$	$\frac{99+28c_{2\theta}+c_{4\theta}}{4(1+c_\theta)}$	$\mathrm{i}\,(-102+105c_\theta-70c_{2\theta}+7c_{3\theta}-4c_{4\theta})\csc\theta$	$\frac{75+107c_\theta+326c_{2\theta}-268c_{3\theta}+47c_{4\theta}-31c_{5\theta}}{1-c_{2\theta}}$
Sum	0	0	0

Table S2: Exact energy cancellations at each order of (E^4, E^3, E^2) in our double-copied four-graviton scattering amplitude (22). An common overall factor $(\kappa^2 m^2/2048)$ in each amplitude is not displayed for simplicity.

Next, we extend the conventional BCJ double-copy method [8][9][10] to the 3d massive gauge boson and graviton amplitudes, where we can construct the desired kinematic numerators (S23) in the gauge boson amplitude which obey the Jacobi identity. Thus, we apply the color-kinematics duality to the four-point massive gauge boson scattering amplitude in Eq.(20) (main text) with the numerators (S23), and construct the four-point massive graviton scattering amplitude of the TMG theory which can be summarized in the following compact form in terms of the Mandelstam variable $s_0\,(=s-4m^2)$ and the scattering angle θ:

$$\begin{aligned}\mathcal{M}[4h_{\rm P}] &= \frac{\kappa^2}{16}\left(\frac{\mathcal{N}_s'^2}{s-m^2}+\frac{\mathcal{N}_t'^2}{t-m^2}+\frac{\mathcal{N}_u'^2}{u-m^2}\right)\\ &= -\frac{\kappa^2 m^2(P_0+P_2c_{2\theta}+P_4c_{4\theta}+P_6c_{6\theta}+\bar{P}_2s_{2\theta}+\bar{P}_4s_{4\theta}+\bar{P}_6s_{6\theta})\csc^2\theta}{4096\,(3+\bar{s}_0)(4+\bar{s}_0)^{3/2}(2+\bar{s}_0-\bar{s}_0c_\theta)(2+\bar{s}_0+\bar{s}_0c_\theta)}\,,\end{aligned} \tag{S24}$$

where $(P_j,\,\bar{P}_j)$ are the polynomial functions of the dimensionless Mandelstam variable $\bar{s}_0=s_0/m^2$,

$$\begin{aligned}P_0 &= -4(7992\bar{s}_0^2+4767\bar{s}_0^3+692\bar{s}_0^4)(4+\bar{s}_0)^{\frac12}\,,\\ P_2 &= (-221184-304128\bar{s}_0-114048\bar{s}_0^2-10928\bar{s}_0^3+505\bar{s}_0^4)(4+\bar{s}_0)^{\frac12},\\ P_4 &= 4(55296+45312\bar{s}_0+13208\bar{s}_0^2+1563\bar{s}_0^3+58\bar{s}_0^4)(4+\bar{s}_0)^{\frac12},\\ P_6 &= -(98304+57344\bar{s}_0+11264\bar{s}_0^2+832\bar{s}_0^3+17\bar{s}_0^4)(4+\bar{s}_0)^{\frac12},\\ \bar{P}_2 &= \mathrm{i}(-442368-663552\bar{s}_0-300672\bar{s}_0^2-46048\bar{s}_0^3+540\bar{s}_0^4+475\bar{s}_0^5),\\ \bar{P}_4 &= \mathrm{i}4(110592+104448\bar{s}_0+36880\bar{s}_0^2+5828\bar{s}_0^3+372\bar{s}_0^4+5\bar{s}_0^5),\\ \bar{P}_6 &= -\mathrm{i}(196608+139264\bar{s}_0+35328\bar{s}_0^2+3776\bar{s}_0^3+148\bar{s}_0^4+\bar{s}_0^5).\end{aligned} \tag{S25}$$

Then, parallel to Eq.(26) of the main text, we make the high energy expansion in terms of $1/\bar{s}_0$, and obtain the following leading nonzero graviton scattering amplitude:

$$\mathcal{M}_0'[4h_{\rm P}] = -\frac{\mathrm{i}\kappa^2 m}{2048}\,s_0^{\frac12}(494c_\theta+19c_{3\theta}-c_{5\theta})\csc^3\theta, \tag{S26}$$

which scales as $\mathcal{O}(mE)$ and takes the same form as Eq.(26) except the replacement $s^{1/2}\!\rightarrow s_0^{1/2}$. We have further verified the exact energy cancellations at each order of $\bar{s}_0^{n/2}$ with $n=4,3,2,$ which are summarized by the present Table S2 in parallel to the Table 2 of the main text.

References

[1] Y.-F. Hang and H.-J. He, "Structure of Kaluza-Klein Graviton Scattering Amplitudes from Gravitational Equivalence Theorem and Double-Copy," Phys. Rev. D 105 (2022) no.8, 084005 [arXiv:2106.04568 [hep-th]].

[2] B. Binegar, "Relativistic Field Theories in Three-dimensions," J. Math. Phys. 23 (1982) 1511.

[3] For a review, G. V. Dunne, "Aspects of Chern-Simons theory", arXiv:hep-th/9902115 [hep-th].

[4] R. Jackiw and V. P. Nair, "Relativistic wave equations for anyons", Phys. Rev. D 43 (1991) 1933.

[5] R. Banerjee, B. Chakraborty and T. Scaria, "Polarization vectors, doublet structure and Wigner's little group in planar field theory," Int. J. Mod. Phys. A 16 (2001) 3967 [arXiv:hep-th/0011011 [hep-th]].

[6] R. D. Pisarski and S. Rao, "Topologically Massive Chromodynamics in the Perturbative Regime," Phys. Rev. D 32 (1985) 2081.

[7] Y.-F. Hang, H.-J. He, C. Shen, "Structure of Chern-Simons Scattering Amplitudes from Topological Equivalence Theorem and Double-Copy," JHEP 01 (2022) 153 [arXiv:2110.05399 [hep-th]].

[8] Z. Bern, J. J. M. Carrasco, and H. Johansson, "New relations for gauge-theory amplitudes", Phys. Rev. D 78 (2008) 085011 [arXiv:0805.3993 [hep-th]].

[9] Z. Bern, J. J. M. Carrasco, H. Johansson, "Perturbative Quantum Gravity as a Double Copy of Gauge Theory", Phys. Rev. Lett. 105 (2010) 061602 [arXiv:1004.0476 [hep-th]].

[10] For a review, Z. Bern, J. J. M. Carrasco, M. Chiodaroli, H. Johansson, and R. Roiban, "The Duality Between Color and Kinematics and its Applications", [arXiv:1909.01358 [hep-th]].
